# Hsp90 and phosphorylation of the Slt2(Mpk1) MAP kinase activation loop are essential for catalytic, but not non-catalytic, Slt2-mediated transcription in yeast

**DOI:** 10.1007/s12192-022-01274-0

**Published:** 2022-04-14

**Authors:** Stefan H. Millson, Andrew W. Truman, Peter W. Piper

**Affiliations:** 1 School of Life Sciences, University of Lincoln, Brayford Pool, Lincoln, LN6 7DL UK; 2 Department of Biological Sciences, The University of North Carolina at Charlotte, Charlotte, NC 28223 USA; 3 Department of Molecular Biology and Biotechnology, University of Sheffield, Western Bank, Sheffield, S10 2TN UK

**Keywords:** Slt2(Mpk1), MAP kinase, Hsp90, Transcription activation, Yeast

## Abstract

In yeast, the Slt2(Mpk1) stress-activated protein kinase directs the activation of two transcription factors, Rlm1 and Swi4/Swi6, in response to cell wall stress. Rlm1 is activated through a phosphorylation by Slt2, whereas the Swi4/Swi6 activation is noncatalytic and triggered by the binding of phosphorylated forms of both Slt2 and a catalytically inactive pseudokinase (Mlp1). Previous studies have delineated a role for the molecular chaperone Hsp90 in the activation of Slt2, but the involvement of Hsp90 in these events of catalytic versus non-catalytic cell integrity signaling has remained elusive. In cells lacking Mlp1, the Hsp90 inhibitor radicicol was found to inhibit the Slt2-mediated catalytic activation of Rlm1, but not the noncatalytic activation of Swi4/Swi6. Mutation of residues in the TEY motif of the Slt2 activation loop strongly impacted both Hsp90 binding and Rlm1-mediated transcription. In contrast, many of these same mutations had only modest effects on Swi4/6 (Slt2-mediated, non-catalytic) transcription, although one that blocked both the Slt2:Hsp90 interaction and Rlm1-mediated transcription (E191G) triggered a hyperactivation of Swi4/6. Taken together, our results cement the importance of the Slt2 activation loop for both the binding of Hsp90 by Slt2 and the catalytic activation of cell integrity signaling.

## Introduction

Much of the kinome of eukaryotic cells requires the Cdc37 and Hsp90 molecular chaperones for its stabilization and activation. Many, though apparently not all, of these protein kinases are therefore considered “clients” of the Cdc37/Hsp90 chaperone system. In many instances, the activation of a protein kinase by these chaperones involves the initial formation of Cdc37-bound state with low binding affinity for Hsp90, which then progresses to a later stage complex in which the kinase client is now in tight association with Hsp90 (Pearl 2005; Vaughan et al. 2008).

Key in the highly interactive protein kinase signaling networks of cells are the mitogen-activated protein (MAP) kinase cascades, where 3 protein kinases stimulate each other in series (MAP kinase kinase kinase ➔ MAP kinase kinase ➔ MAP kinase). Based on the components of these signaling cascades that are destabilized upon Hsp90 inhibitor treatment, it has been proposed that the upstream kinases generally require the Hsp90 chaperone function whereas the terminal MAP kinases are not clients of Hsp90 (Tajsharghi et al. 2007; Citri et al. 2006). Many MAP kinases are already in an active form when expressed in bacteria in the absence of eukaryotic forms of Hsp90 (Khokhlatchev et al. 1997; Heise and Cobb 2006). In addition, some MAP kinases are activated, not inhibited, when cells are treated with Hsp90 inhibitors (e.g. ERK1/2 Citri et al. 2004; Koga et al. 2006)), while others are relatively insensitive to such inhibitor treatment (e.g. JNK1 see (Prince and Matts 2005) for literature). There are though exceptions. High activity of the terminal kinase of the *Saccharomyces cerevisiae* cell integrity signaling pathway, Slt2(Mpk1) requires Cdc37 and Hsp90 (Millson et al. 2005; Truman et al. 2007; Hawle et al. 2007), with Slt2 displaying a reduced activity and stability in *cdc37* mutants (Hawle et al. 2007). Conversely, the inactive ERK5 MAP kinase of mammalian cells resides in the cytosol as a complex with Hsp90, its activation leading to a dissociation from Hsp90, autophosphorylation and translocation to the nucleus (Erazo et al. 2013, 2020).

It is becoming clear that the phosphorylation status of a protein kinase can sometimes determine whether it associates with Hsp90. In yeast strong Hsp90 binding to either the native Slt2 or a heterologously expressed human ERK5 occurs in response to the activation phosphorylation of these MAP kinases by the endogenous upstream MAP kinase kinase (Mkk1/2) (Millson et al. 2005; Truman et al. 2006). This is in stark contrast to the situation in mammalian cells where the activation phosphorylation of ERK5 causes ERK5 to dissociate from Hsp90 (Erazo et al. 2013, 2020); also, the AGC kinases Akt and cPKC, in which activation phosphorylation of a conserved turn motif by mTORC2 generates an independence from Hsp90 chaperoning (Facchinetti et al. 2008).

Slt2 activates two transcription factors in yeast, Rlm1 and Swi4 (the latter a component of the Swi4/Swi6 (SBF) complex activated at the start point of the yeast cell cycle (reviewed in (Levin 2005)). Activation of Rlm1 is an Slt2-catalysed process, involving the direct phosphorylation of this transcription factor by the Mkk1/2-phosphorylated, catalytically active Slt2 (Jung et al. 2002; Garcia et al. 2004). Swi4/Swi6 activation is noncatalytic, triggered by the binding of the Mkk1/2-phosphorylated forms of both Slt2 and a catalytically inactive pseudokinase, Mlp1 (Kim et al. 2008). When expressed heterologously in yeast the human ERK5 is also able to execute the latter, noncatalytic activation of Swi4/Swi6 (Kim et al. 2008). Here we have used the availability of well-characterised gene reporters of Rlm1 and Swi4/Swi6 activity (Kim et al. 2008) to probe the requirement for Hsp90 in these events of catalytic versus noncatalytic (namely Rlm1 versus Swi4/Swi6) activation by Slt2. We have also analysed the roles of individual residues within the activation loop TEY motif of Slt2 for the activities of Rlm1 and Swi4/Swi6, as well as the association of Slt2 with Hsp90. Our data reveals that the catalytic activation of Rlm1 by Slt2 is Hsp90-dependent, whereas the noncatalytic activation of Swi4/Swi6 can still occur when Hsp90 is inhibited. Slt2 MAP kinase appears therefore to need Hsp90 for the former, but not the latter, function.

## Materials and methods

### Yeast strains and growth

The yeast strains used for this study are listed in Table 1. Conditions of yeast growth and radicicol treatment were as previously described (Truman et al. 2007). The *URA3-*based *PRM5-lacZ* and *FKS2-lacZ* vectors (Kim et al. 2008), multicopy plasmid YEpSLT2 (Kamada et al. 1996); also, the vectors for His-tagged E33A mutant Hsp82 expression (Millson et al. 2005) were as previously described.

**Table 1:** The yeast strains used in this study

Strain	Genotype	Reference
W303-1a	MATa *ura1-1 trp1-1 leu2-3,112 his3-11 ade2-1 can1-100 ssd1-3 ybp1-1*	
FL-HA	W303-1a SLT2(1–484)::3HA-*TRP1*	This study
(1–464)-HA	W303-1a SLT2(1–464)::3HA-*TRP1*	This study
(1–370)-HA	W303-1a SLT2(1–370)::3HA-*TRP1*	This study
BY4741	a *his3-Δ1 leu2-Δ0 met15-Δ ura3-Δ0*	Research Genetics
BY4741*mlp1Δ*	BY4741*mlp1ΔkanMX4*	Research Genetics
BY4741*rlm1Δ*	BY4741*rlm1ΔkanMX4*	Research Genetics
BY4741*swi4Δ*	BY4741*swi4ΔkanMX4*	Research Genetics
BY4741*mlp1Δ slt2Δ*	BY4741*mlp1ΔkanMX4 slt2ΔhphMX4*	This study
SHM-WT	BY4741*mlp1ΔkanMX4 SLT2-HA::hphMX4*	This study
SHM-T190A	BY4741*mlp1ΔkanMX4 slt2(T190A)-HA::hphMX4*	This study
SHM-E191G	BY4741*mlp1ΔkanMX4 slt2(E191G)-HA::hphMX4*	This study
SHM-Y192F	BY4741*mlp1ΔkanMX4 slt2(Y192F)-HA::hphMX4*	This study
SHM-TA/YF	BY4741*mlp1ΔkanMX4 slt2(TA/YF)-HA::hphMX4*	This study
PJ694a	MATa *trp1-901 leu2-3 112 ura3-52 his3-200 gal4 gal80 LYS2::GAL1-HIS3 GAL2-ADE2 met2::GAL7-lacZ*	

Amino acid changes were introduced into the *SLT2-3xHA* gene of YEpSLT2 by QuickChange mutagenesis (Stratagene). For genomic replacement of the coding sequences of the native chromosomal *SLT2* gene in BY4741*mlp1Δ* (Table 1) by the wild type and mutant *SLT2-3xHA* alleles of these YEpSLT2-derived vectors, the following approach was adopted. Each *SLT2-3xHA* gene was first reamplified from the relevant YEpSLT2-derived vector using primers: CTCTCGGATCCATGGCTGATAAGATAGAGAGG and GAGAGGGCGCGCCCTAAAAATATTTTCTATC (*Bam*H1 and *Asc*II sites underlined); then cloned into *Bam*H1 + *Asc*II-cleaved pAG32 (Goldstein and McCusker 1999). Next, a fragment containing both the *SLT2* and *HygB* genes was PCR amplified from this pAG32-based plasmid, using primers GAAGGGCGTGTATAACAATTCTGGGAG*ATGGCTGATAAGATAGAGAGG* and CTTACATCTATGGTGATTCTATACTTCCCCGG*CGCTAACATTTGATTAAAATA*G (homology to pAG32 in italics). This was then used to transform strain BY4741*mlp1Δ* (Table 1) to hygromycin resistance. W303-1a-based strains expressing either the full-length or truncated, C-terminally 3xHA-tagged forms of Slt2 (Table 1) were constructed by *TRP1-HA* cassette (Longtine et al. 1998) integration. Colony PCR was used to confirm correct cassette integration at the *SLT2* locus, as well as the confirmation of amino acid changes by dye-terminator sequencing.

### Analysis of Slt2 levels and phosphorylation

Total protein extracts were prepared, then analysed for levels of Hsp90 and Slt2 MAP kinase essentially as described previously (Millson et al. 2005; Truman et al. 2006, 2007). The antisera used were as follows: for the analysis of dually phosphorylated (Thr^190^/Tyr^192^)-Slt2, a commercial antibody raised against dually phosphorylated (Thr^202^/Tyr^204^)-p44/42 MAP kinase (New England Biolabs) that recognises the dually phosphorylated (Thr^190^/Tyr^192^)-Slt2 MAP kinase in yeast cell extracts (Martin et al. 2000); for Slt2-3xHA analysis, a HA-Tag (6E2) mouse monoclonal antibody (Cell Signalling); for Hsp82-His_6_ analysis, a monoclonal anti-tetraHis antibody (Qiagen). Slt2-HA was isolated by immunoprecipitation using anti-HA immunoglobulin G-coated agarose beads (Sigma).

### Transcription factor activity measurements

β-galactosidase assays of promoter-*lacZ* fusion expression were as previously described (Kim et al. 2008), data points being of duplicate experiments and the mean and SD of eight individual assays.

### Yeast two-hybrid analysis

To analyse the effects of single Slt2 mutations on yeast two-hybrid (Y2H) interactions, these mutations were introduced into a previously described Slt2-*GAL4* activator domain (AD) fusion “bait” vector (Millson et al. 2005), confirmed by dye-terminator sequencing, and then transformed into PJ694a (Table 1) for an analysis of their effects on Y2H interactions with Hsp82-*GAL4* DNA binding domain (BD) and Hsp82(E33A)-BD “bait” fusions (Millson et al. 2005; Truman et al. 2006). Manipulations of Y2H “bait” and “prey”-expressing transformants, also automated measurements of β-galactosidase activity due to the interaction-responsive, *GAL7* promoter-regulated *LacZ* gene of PJ694 were as previously described (Millson et al. 2004, 2005). β-galactosidase activity data shown being the mean and SD of eight individual assays on separate aliquots of each culture.

## Results

### The C-terminus of Slt2 is dispensable for the heat induction of FKS2

In previous work, we had found that the binding of Hsp90 by Slt2 is stress-dependent, occurring in response to the dual phosphorylation of Slt2 at T190 and Y192 (Millson et al. 2005; Truman et al. 2006). Slt2, together with the human ERK5, are unusual amongst MAP kinases in possessing an unusually long C-terminal sequence extension to the protein kinase module. Expression of a C-terminally truncated Slt2 that lacks this C-terminal sequence extension (amino acids 1–370 comprising just the protein kinase domain) rescues some, but not all, *slt2Δ* mutant phenotypes in yeast (Soler et al. 1995; Kirchrath et al. 2000; Truman et al. 2006). Previously, we had found that just the N-terminal 1–326 region of Slt2 (a fragment that is catalytically inactive as it lacks the α1L14/α2L14 C-lobe region unique to MAP kinases) was sufficient for the stress-induced interaction with Hsp90, indicating that the association with Hsp90 does not require the long C-terminal region of this MAP kinase (Millson et al. 2005).

**Fig. 1:**
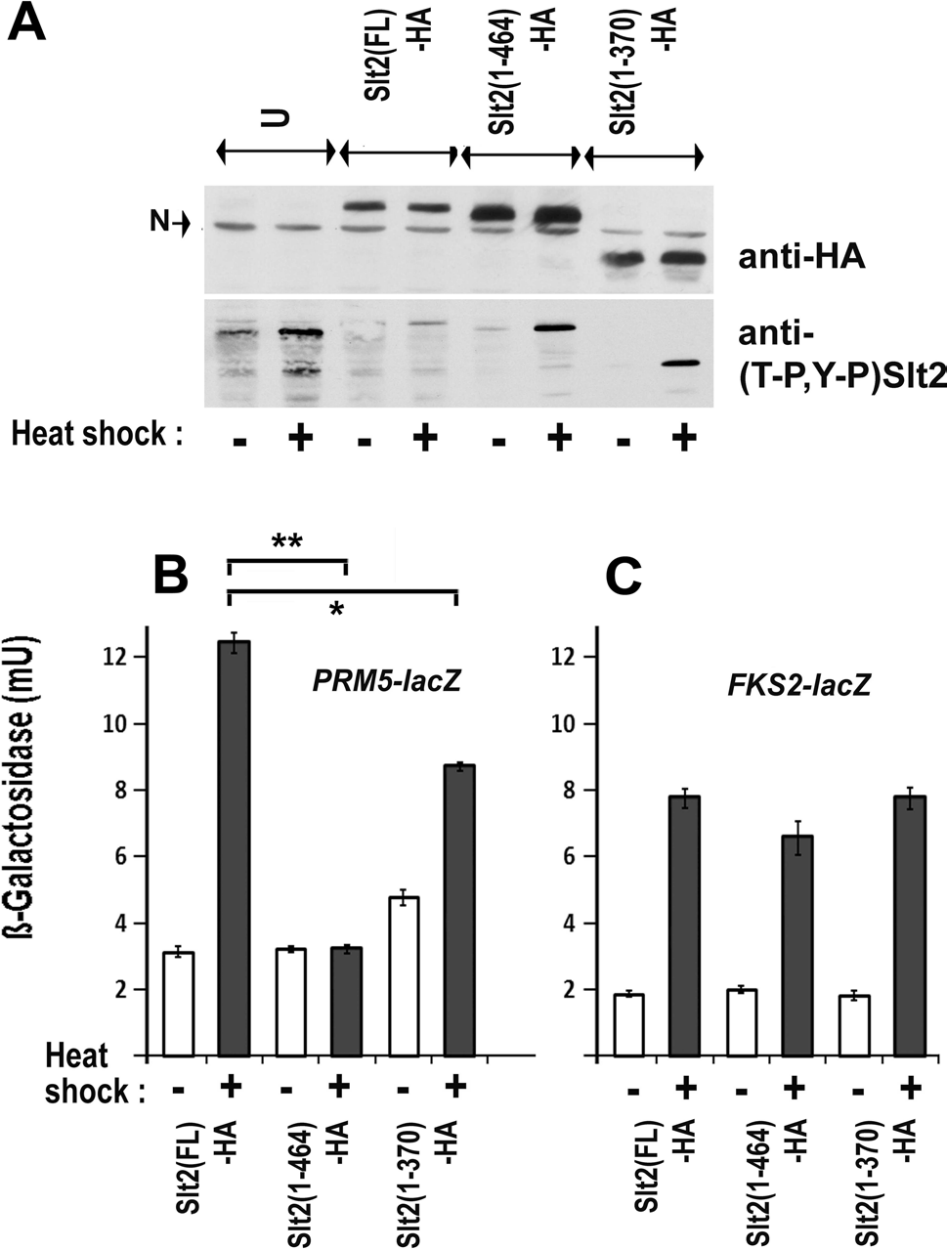
**A** Analysis of Slt2-HA, also dually phosphorylated Slt2 ((T-P,Y-P) Slt2) in total cell extracts prepared from strains W303-1a (U); FL-HA, (1–464)-HA and (1–370)-HA (Table 1); before ( −) and after ( +) exposure to a 1 h 37 °C heat shock. Ten-microgram total cell protein was loaded in each lane, the nonspecific band detected by the anti-HA antiserum (N) effectively acting as a loading control. **B**, **C** Measurements of *PRM5-lacZ* (**B**) and *FKS2-lacZ* (**C**) expression in these cells of FL-HA, (1–464)-HA and (1–370)-HA under the same conditions (mean and SD of 8 assays on each culture; two-tailed *t* test; * = *p* < 0.05; ** = *p* < 0.01)

To determine whether truncated forms of Slt2 could be used to investigate the Hsp90 dependence of Rlm1 and Swi4/Swi6 activity, transcription of *PRM5-LacZ* and *FKS2-LacZ* reporter genes was initially studied in cells that express either a full-length, or truncated (1–464 or 1–370) forms of a 3xHA epitope tagged Slt2 (Fig. 1B). Heat activation of *PRM5-LacZ* was abolished with the loss of just the C-terminal 20 amino acids in Slt2 ((1–464)-HA), although it was partially restored in the cells expressing (1–370)-HA Slt2 (Fig. 1B). In contrast, the noncatalytic activation of *FKS2-LacZ* by heat was largely unaffected by the loss of these C-terminal regions of Slt2 (Fig. 1C), despite a caffeine-induced phosphorylation (at Ser423) within this region having the capacity to prevent the Slt2:Swi4 association (Truman et al. 2009). Importantly, for the purposes of our study, the measurements in Fig. 1 revealed the need to use cells expressing a full-length Slt2 when monitoring any effects of the pharmacological inactivation of Hsp90 on Rlm1 activity, despite just the N-terminal region of this MAP kinase retaining a physical interaction with Hsp90 (Millson et al. 2005).

### The Hsp90 inhibitor radicicol suppresses Rlm1 but not Swi4/6-mediated transcription

We next investigated how loss of Hsp90 function would affect the Slt2-directed activation of these *PRM5-lacZ* and *FKS2-lacZ* fusion genes, reporters of the activation of Rlm1 or Swi4/Swi6 respectively (Kim et al. 2008). We transformed these reporters into a yeast lacking Mlp1 prior to analysing how their induction was impacted by the Hsp90 inhibitor radicicol (Mkk1/2-phosphorylation of Slt2 still occurs in radicicol-treated cells (Truman et al. 2007)). The radicicol concentration used (100 μM) was that needed to achieve a total growth arrest of this strain, while the use of an *mlp1Δ* genetic background eliminated any confounding influences of the Mlp1 pseudokinase.

**Fig. 2:**
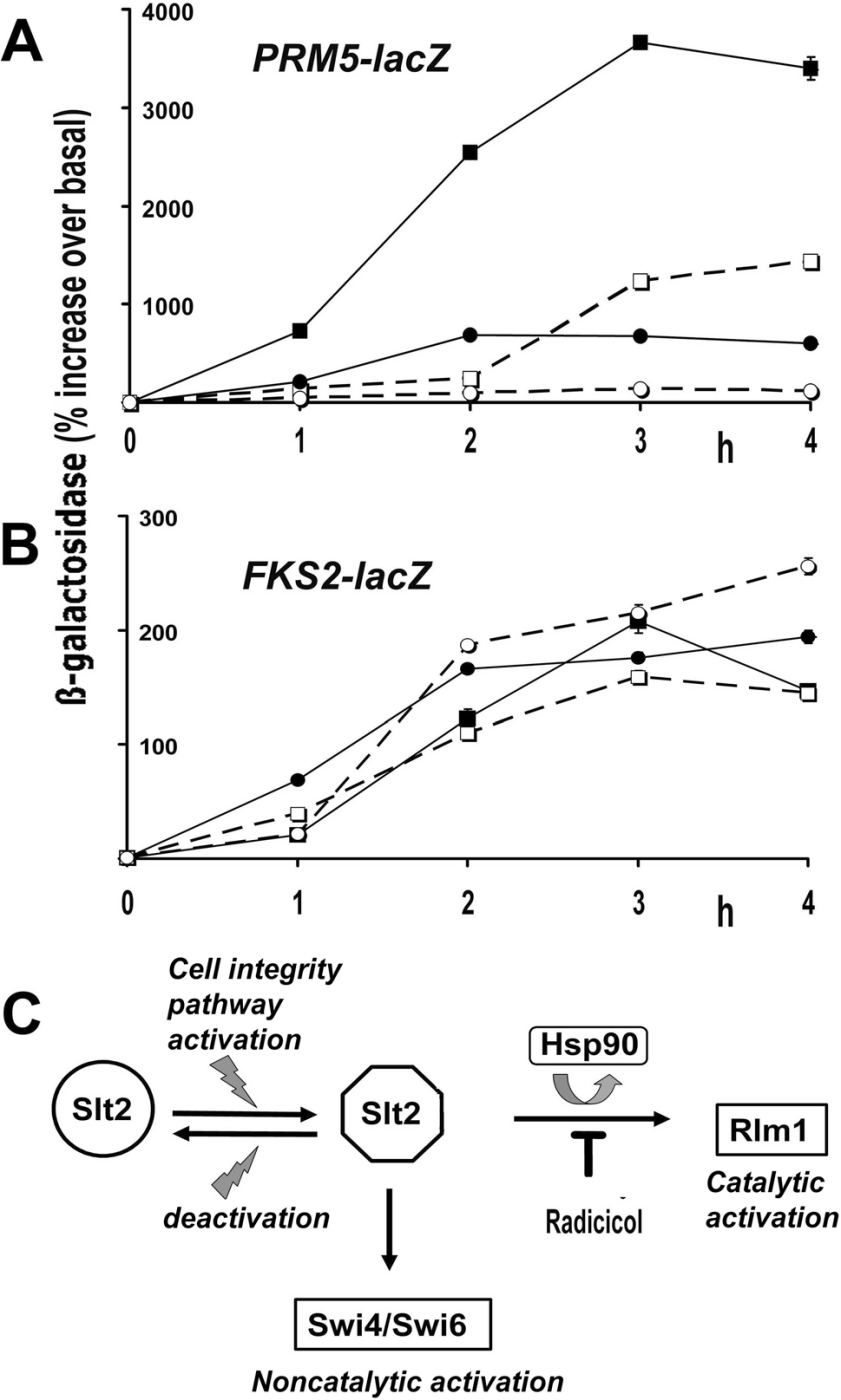
Time course of **A** *PRM5-lacZ* and **B** *FKS2-lacZ* induction in BY4741*mlp1Δ* in the presence of either radicicol (❑,❍) or vehicle DMSO (■,●). Induction was by either 5 mM orthovanadate treatment at 25 °C alone (■,❑), or a combined 5 mM orthovanadate treatment and heat shock from 25 to 37 °C (●,❍). Data is expressed relative to the activity of β-galactosidase in these cultures at time zero, prior to the stress application. **C** These effects of radicicol indicate that Hsp90 is needed for the catalytic activation of Rlm1 but not the noncatalytic activation of Swi4/Swi6

In preliminary experiments, we found that we could obtain the highest levels of Slt2-catalysed Rlm1 (*PRM5-lacZ*) induction at 25 °C using 5 mM orthovanadate as an inducer. Orthovanadate is a potent, though relatively slow, inducer of Slt2 activity (Jimenez-Sanchez et al. 2007). At this temperature, the orthovanadate induction of Rlm1 was substantially, though not completely, abolished by radicicol (Fig. 2A). In contrast, there was no inhibition of the Slt2-dependent, noncatalytic (Kim et al. 2008) activation of Swi4/Swi6 (*FKS2-lacZ*) under these conditions of radicicol treatment (Fig. 2B). Lower levels of Rlm1 induction were apparent when the applied stress was a combined 5 mM orthovanadate treatment and heat shock to 37 °C, but this induction was now totally abolished by radicicol (Fig. 2A). Swi4/Swi6 induction under these same conditions of a combined 5 mM orthovanadate treatment and a heat shock to 37 °C was again unaffected by radicicol, being comparable to that seen with the orthovanadate treatment at 25 °C alone (Fig. 2B).

These results indicate that Slt2 catalytic activity—conveniently monitored in vivo as the activity of Rlm1 (Jung et al. 2002)—is substantially Hsp90-dependent at a moderate temperature, yet completely Hsp90-dependent at 37 °C, whereas the Slt2-directed, noncatalytic activation of Swi4/Swi6 is independent of Hsp90, even at 37 °C.

### Mutation of the TEY phosphorylation motif of Slt2 impacts differently on Rlm1-directed versus Swi4/6-directed transcription

Activation of MAP kinases occurs when the upstream MAP kinase kinase catalyses dual Thr/Tyr phosphorylation of a TXY motif in the MAP kinase activation loop (X corresponding to a Glu in mammalian ERK1/2/5 and yeast Slt2/Kss1/Fus3, Pro in JNK and Gly in p38/Hog1 (Tanoue and Nishida 2003)). During activation, Slt2 is initially phosphorylated at Y192. A recent study has found that this Y192 phosphorylation is important for the subsequent phosphorylation at T190, the latter being the key event for high catalytic activity (Gonzalez-Rubio et al. 2021). To understand the relative importance of the individual amino acids within the Slt2 TEY motif for catalytic versus noncatalytic transcription, we analyzed the effects of mutating each of these three residues in turn. Mutations T190A and Y192F are conservative substitutions that abolish phosphorylation at T190 or Y192 respectively. E191G changes this motif from TEY (as found in the mammalian ERK1/2/5 and yeast Slt2/Kss1/Fus3 MAP kinases) to TGY (as found in MAP kinases of the p38/Hog1-family). These three single mutations (T190A, E191G, and Y192F), as well as the double T190A Y192F (TA/YF) mutation, were each introduced at the chromosomal *SLT2* locus of strain BY4741*mlp1Δ* (Table 1), thus enabling the expression of the corresponding mutant form of full-length Slt2 (3xHA-tagged at its C-terminus) in place of the wild type Slt2.

**Fig. 3:**
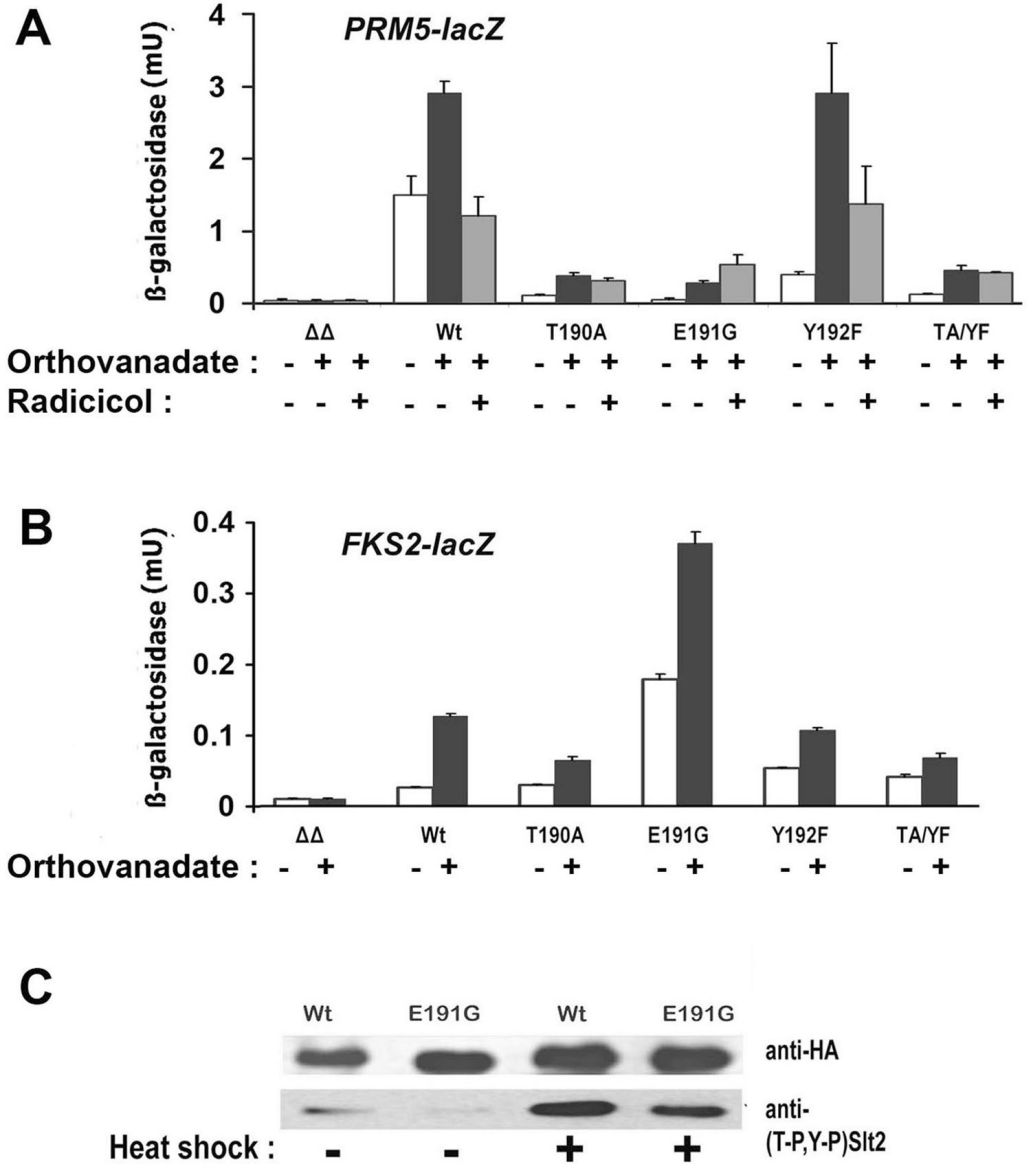
**A** *PRM5-lacZ* and **B** *FKS2-lacZ* expression in BY4741*mlp1Δ* cells that either µtotally lacked Slt2 (ΔΔ) or contained the wild type (Wt) or indicated mutant forms of Slt2-HA; at 25 °C without or with a prior 4 h treatment with 5 mM orthovanadate treatment (in **A** also with or without radicicol added at the time of the orthovanadate application). **C** Analysis of dually phosphorylated Slt2-HA in SHM-WT and SHM-E191G cultures maintained at 25 °C, either before ( −) or after ( +) this treatment with 5 mM orthovanadate. Ten-microgram total cell protein was loaded in each lane

Next, we analysed the impact of these mutations on *PRM5-lacZ* and *FKS2-lacZ* induction in 25 °C cultures exposed to a 5 mM orthovanadate treatment. Expression of the *slt2*(*T190A*), *slt2*(*E191G*), and *slt2*(*TA/YF*) alleles resulted in substantially lowered basal and induced Rlm1 activity (Fig. 3A). It is noteworthy however that *slt2*(*T190A*), *slt2*(*E191G*), and *slt2*(*TA/YF*) still enabled a limited *PRM5-lacZ* induction, demonstrably higher than that of BY4741*mlp1Δslt2Δ* cells devoid of any Slt2. The levels of induced Rlm1 activity were essentially unaltered by *slt2*(*Y192F*), even though the basal Rlm1 activity at 25 °C prior to the application of stress was lower in the cells containing this *slt2(Y192F)* allele (Fig. 3A). Induction of Rlm1 activity in these *slt2*(*Y192F*) cells was reduced, though not abolished in the presence of radicicol (Fig. 3A).

Basal and induced levels of *FKS2-lacZ* (Swi4/Swi6) expression were only moderately reduced by the *slt2*(*T190A*), *slt2*(*TA/YF*) and, to lesser extent, the *slt2*(*Y192F*) alleles (Fig. 3B). Unexpectedly, *slt2*(*E191G*) resulted in basal and induced levels of Swi4/Swi6 activity that were higher than in the cells expressing the wild type Slt2 (Fig. 3B). To examine whether the greatly reduced Rlm1 activation with *slt2*(*E191G*) expression could be attributed to the loss of Slt2 phosphorylation, we assessed Slt2 phosphorylation in wild type and *slt2*(*E191G*) cells both before and after this orthovanadate treatment by western blotting (Fig. 3C). This revealed that the Slt2(E191G) protein was still becoming substantially phosphoryated with stress, indicating that the observed loss of Rlm1 activity in *slt2*(*E191G*) cells (Fig. 3A) is not due merely to a total lack of Slt2 phosphorylation.

### Mutations within the TEY activation loop motif compromise the interaction between Hsp90 and Slt2

**Fig. 4:**
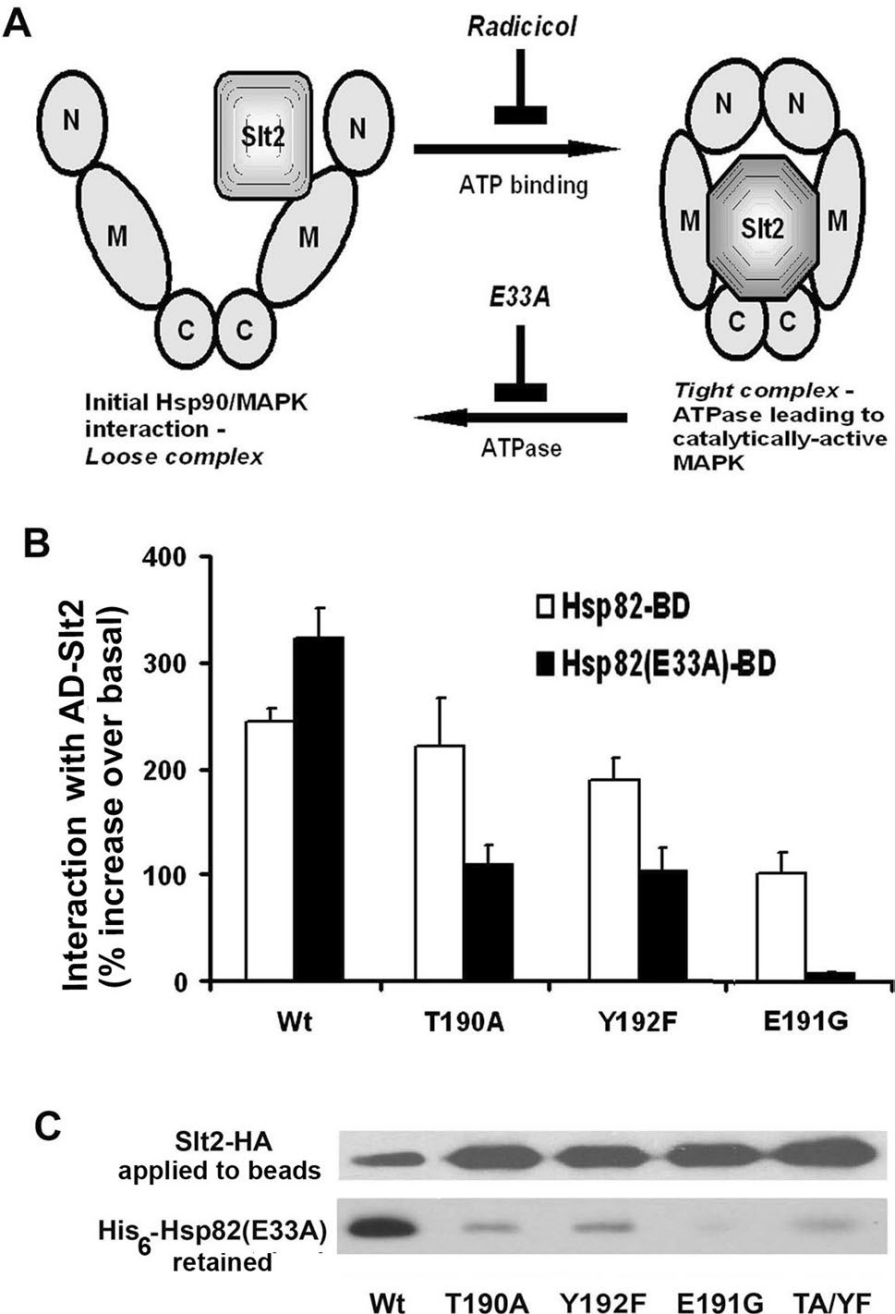
**A** Radicicol inhibits the ATP-mediated formation of a tight Hsp90 client complex, whereas the E33A Hsp90 mutation inhibits the ATPase activity of this chaperone and release of the activated client. **B** The effects of mutations in the activation loop TEY motif of an AD-Slt2 fusion on heat induced Y2H interaction with Hsp82-BD and Hsp82(E33A)-BD bait fusions (stress applied as in earlier studies Millson et al. 2005; Truman et al. 2006)). **C** His_6-_Hsp82(E33A) retained by the Slt2-HA bound to anti-HA immunoglobulin G-coated agarose beads in extracts from unstressed (25 °C) cultures of SHM-WT, SHM-T190A, SHM-E191G, SHM-Y192F, and SHM-TA/YF

Quantitative measurements of in vivo Y2H interaction strength have shown that the Hsp90 binding by Slt2 is abolished with double T190A,Y192F (TA/YF) mutation of Slt2 yet reinforced by a Hsp90 mutation (E33A) that arrests the chaperone ATPase cycle (Fig. 4A) (Millson et al. 2005; Truman et al. 2006). Extending this study, we introduced the T190A, E191G, and Y192F mutations into the AD-Slt2 Y2H “prey” fusion and then measured how, in the Y2H system, they affected the interactions of this AD-Slt2 with the wild type and a E33A mutant form of the Hsp90-BD “bait” fusion. Of these mutational changes, only E191G substantially weakened the heat-reinforcement of the interaction with Hsp90-BD (a functional form of Hsp90 (Millson et al. 2003)) (Fig. 4B). It is noteworthy however that both T190A and Y192F led to a weakening, rather than the usual reinforcement, of AD-Slt2:Hsp90-BD interaction with the introduction of the E33A mutation into this Hsp90-BD bait, while E191G totally abolished any reinforcement of the AD-Slt2: Hsp90(E33A)-BD interaction by stress (Fig. 4B).

To further validate these Y2H results, we performed anti-HA bead retention experiments on extracts of cells expressing both HA-tagged Slt2 (T190A, Y192F, E191G, and TA/YF versions) and His-tagged Hsp90(E33A). Consistent with the quantitative measurements of in vivo interaction strength (Fig. 4B), all these the activation loop mutant forms of Slt2-HA displayed a greatly diminished binding of Hsp90(E33A)-His (Fig. 4C).

### Slt2 activation loop mutants display unique phenotypic fingerprints

**Fig. 5:**
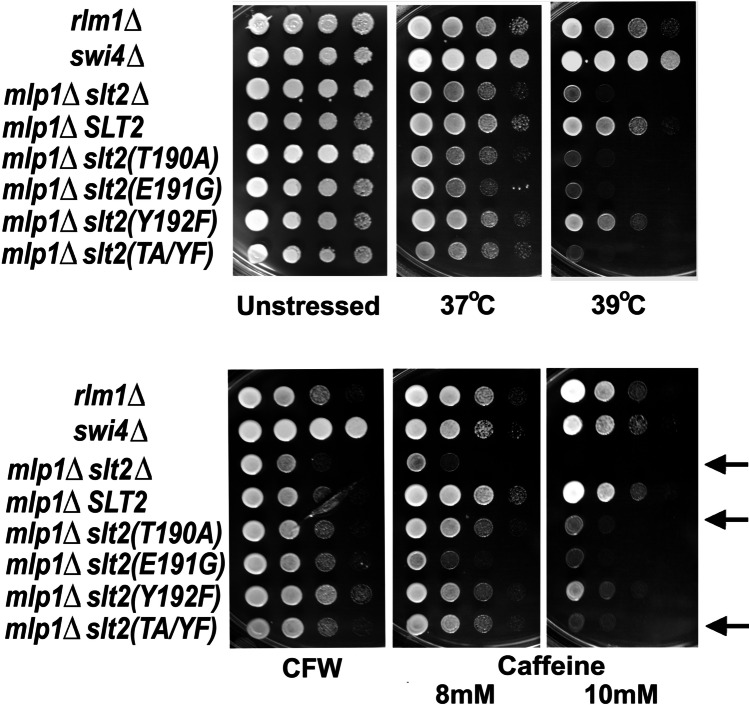
Strains of BY4741 genetic background, spotted in 1:10 dilution series on YPD and grown 3 days at 30, 37 or 39 °C (upper images), or 30 °C in the presence of 25ug/ml CFW, 8 mM caffeine or 10 mM caffeine (lower images). Note how *slt2*(*T190A*) and *slt2*(*TA/YF*) expression provide a limited rescue of the CFW and caffeine sensitivities apparent in *mlp1Δslt2Δ* cells (arrowed)

Total loss of Slt2 activity is associated with a number of phenotypes in yeast, including temperature- and caffeine-sensitivity (Watanabe et al. 1995; Kamada et al. 1996; Martin et al. 2000), lack of glycerol growth (Watanabe et al. 1995), and a sensitivity to compounds that affect the cell wall (such as SDS and calcofluor white (CFW) (Kirchrath et al. 2000; de Nobel et al. 2000). Deletion of Rlm1 and Swi4 leads to different phenotypes. The loss of Rlm1 causes caffeine sensitivity (less marked than that of *slt2Δ*) but it has no impact on high-temperature growth (Watanabe et al. 1995). In contrast, the loss of Swi4 leads to a slight temperature sensitivity but no sensitivity to caffeine (Madden et al. 1997; Truman et al. 2009). We investigated some of these Slt2-dependent phenotypes in our *mlp1Δ* cells expressing TEY motif mutant forms of Slt2 since, as far as we are aware, they had not previously been analyzed in a genetic background that lacks the Mlp1 pseudokinase. As in earlier studies (Lee et al. 1993), these phenotypes were only weakly affected by *slt2*(*Y192F*) (Fig. 5), consistent with the relatively modest effects of this mutation on Rlm1 and Swi4/6 activity (Fig. 3). However, *slt2*(*T190A*), *slt2*(*E191G*), and *slt2*(*TA/YF*) were as temperature-sensitive, and almost as sensitive to caffeine and CFW, as the *mlp1Δ slt2Δ* cells (Fig. 5) consistent with the almost total lack of Rlm1 and reduced Swi4/6 activity in these cells (Fig. 3).

Interestingly, *slt2*(*T190A*), *slt2*(*TA/YF*), and—to a lesser extent—*slt2*(*E191G*) still provided a limited rescue of the caffeine and CFW sensitivity of *mlp1Δ slt2Δ* cells (Fig. 5), a finding consistent with these alleles still allowing low levels of *PRM5-lacZ* and *FKS2-lacZ* expression (Fig. 3A, B).

## Discussion

Slt2 MAP kinase regulates two major transcription factors in yeast, Rlm1 (activated catalytically by direct Slt2-mediated phosphorylation (Jung et al. 2002)) and Swi4/Swi6 (activated noncatalytically, through the binding of either dually phosphorylated Slt2 or monophosphorylated Mlp1 (Kim et al. 2008)). In this study, we focused on the extents to which these events are Hsp90-dependent; as well as whether the Hsp90:Slt2 interaction is influenced by mutations within the TEY phosphorylation motif of Slt2. Phosphorylation of the activation loop on a MAP kinase serves to relieve the steric inhibition of protein substrate binding to the kinase. In Slt2 it is also the signal for a strong association with Hsp90 (Millson et al. 2005).

In *mlp1Δ* cells, devoid of any confounding influences of the Mlp1 pseudokinase, only the catalytic activation of Rlm1 was subject to inhibition by radicicol, not the noncatalytic activation of Swi4/Swi6 (Fig. 2). This is consistent with Hsp90 being required for the Mkk1/2-phosphorylated Slt2 to attain a state of high catalytic activity, but not for it to execute its noncatalytic activation of Swi4/Swi6 (Fig. 2C). These effects of radicicol reveal that Slt2 is not rendered biologically inert when Hsp90 function is lost. This kinase can therefore be regarded as a “client” of Hsp90 for some, but not all, of its functions (Fig. 2C).

Slt2 is dually Thr^190^/Tyr^192^–phosphorylated with the activation of cell integrity signaling, yet it is the Thr^190^ phosphorylation that promotes its high kinase activity (Gonzalez-Rubio et al. 2021). Consistent with this, we found a much stronger effect of T190A, compared to Y192F, on the Slt2-dependent activities of Rlm1 and Swi4/Swi6 (Fig. 3); as well as stress-sensitivities (Fig. 5) in *mlp1Δ* cells. Nevertheless, both T190A and Y192F—as single mutations—compromise how the E33A mutation in Hsp90 reinforces the interaction between Slt2 and Hsp90 (Fig. 4B, C). The initial Y192 phosphorylation of Slt2, an event key for the subsequent phosphorylation at T190 (Gonzalez-Rubio et al. 2021), may therefore be also important for the formation of a tight complex between Slt2 and Hsp90 (Fig. 4A). There is already precedent for a highly localised change to surface charge distribution acting as the determinant of whether Hsp90 will associate with a protein kinase. In the ErbB1 and ErbB2 receptor tyrosine kinases, a single point mutation—resulting in the loss of a surface negative charge in the αC helix to β4 strand loop of the kinase N lobe—is sufficient to promote the binding of Hsp90 (Xu et al. 2005).

The weakening of in vivo Slt2:Hsp90 association that was caused by E191G was even more marked than that caused by T190A (Fig. 4B). This, together with the Rlm1 activity data (Fig. 3A), highlights the importance of glutamate 191 in the Slt2 activation loop, both for Hsp90 binding and for formation of the catalytically active kinase. Upon phosphorylation of the adjacent Thr^190^ and Tyr^192^ (an event that reinforces Hsp90 binding Millson et al. 2005; Truman et al. 2007) (Fig. 4)), E191 will constitute part of a negative charge cluster. Nevertheless, this glutamic acid alone cannot be viewed as the sole determinant of Hsp90 binding, as a number of MAP kinases that appear not to be clients of Hsp90 also possess a TEY activation loop motif (e.g. ERK1/2; see the “Introduction” section). It is noteworthy that while E191G prevents the catalytic activation of Rlm1 by Slt2, it causes the noncatalytic activation of Swi4/Swi6 to be higher than in the cells with a wild type Slt2 (Fig. 3B). Possible explanations for this might be that the chaperone-free state of this MAP kinase executes its noncatalytic activation of Swi4/Swi6 more efficiently than the chaperone-bound form, or that E191G—by severely weakening Hsp90 binding (Fig. 4)—is acting to increase the former Hsp90-free pool of Slt2. Another explanation might be that E191G is altering the affinity of Slt2 for its Swi4 transcription factor substrate. Such alteration of substrate affinity is seen with the reciprocal TGY to TEY mutation in the p38 MAP kinase (Jiang et al. 1997).

Hsp90 client proteins include diverse regulators of cellular signaling (Prodromou 2012; Theodoraki and Caplan 2012). In yeast, Hsp90 is instrumental in the regulation of diverse stress responses, partly due to its effects on heat shock transcription factor (Truman et al. 2007), calcineurin (Imai and Yahara 2000; Cowen 2008), Slt2 (Millson et al. 2005; Truman et al. 2007) and Sse1 (Shaner et al. 2008). The chaperone dependency of Slt2 is a major underlying cause of many of the phenotypes displayed by yeast mutants in which the essential activities of Cdc37, Hsp90 or heat shock transcription factor are partially compromised (Millson et al. 2005; Truman et al. 2006, 2007; Shaner et al. 2008; Hawle et al. 2007). Though Hsp90 is normally abundant, its levels can become limiting under conditions of stress when there is greater need to stabilize proteins that are prone to misfolding. This is a major reason why mutants partially compromised in the Cdc37/Hsp90 chaperone system display their most marked phenotypes at high temperature (Nathan and Lindquist 1995; Millson et al. 2005; Truman et al. 2006, 2007; Hawle et al. 2007). Equally, it is probable that—in certain instances—the Hsp90-dependence of a client can be partial at low temperature, yet absolute at higher temperature. The radicicol inhibition of Slt2 catalytic activity (Figs. 2 and 3A) indicates that Slt2 may be one such client, with Hsp90 being essential for this MAP kinase to achieve a state of high catalytic activity but nonessential for the low, unregulated basal activity that allows non-activatable, mutant forms of Slt2 to provide a limited rescue of certain phenotypes in *mlp1Δslt2Δ* cells (Fig. 5).
